# Paclitaxel Induces Neurotoxicity by Disrupting Tricarboxylic Acid Cycle Metabolic Balance in the Mouse Hippocampus

**DOI:** 10.1155/2023/5660481

**Published:** 2023-08-05

**Authors:** Xi Liu, Changmeng Cui, Wenxue Sun, Junjun Meng, Jinxiu Guo, Linlin Wu, Beibei Chen, Dehua Liao, Pei Jiang

**Affiliations:** ^1^Department of Pharmacy, Linfen People's Hospital, Linfen, China; ^2^Department of Neurosurgery, Affiliated Hospital of Jining Medical University, Jining, China; ^3^Translational Pharmaceutical Laboratory, Jining First People's Hospital, Jining Medical University, Jining, China; ^4^Department of Oncology, Tengzhou Central People's Hospital, Affiliated to Jining Medical College, Tengzhou, China; ^5^ADFA School of Science, University of New South Wales, Canberra, Australia; ^6^Department of Pharmacy, Hunan Cancer Hospital, Changsha, China; ^7^Translational Pharmaceutical Laboratory, Jining First People's Hospital, Shandong First Medical University Institute of Translational Pharmacy, Jining Medical Research Academy, Jining, China

## Abstract

**Objective:**

It is well known that paclitaxel (PTX)-induced neurotoxicity seriously affects the quality of life of patients and is the main reason for reducing the dose of chemotherapy or even stopping chemotherapy. The current data are limited, and further information is required for practice and verification. The aims of this study were to clarify the molecular mechanism underlying PTX-induced neurotoxicity by combining *in vivo* and *in vitro* metabolomics studies and provide new targets for the prevention and treatment of PTX-induced neurotoxicity.

**Methods:**

In the *in vivo* study, a PTX-induced neurotoxicity mouse model was established by intraperitoneal injection of PTX (6 mg/kg every three days) for two consecutive weeks. After verification by water maze tests and HE staining of pathological sections, hippocampal metabolites were measured and the differential metabolites and related metabolic pathways were identified by multivariate statistical analysis. In the *in vitro* study, we investigated the effects of PTX on mouse hippocampal neuron cells, assessing the concentration and time of administration by MTT assays. After modeling, the relevant metabolites in the TCA cycle were quantified by targeted metabolomics using stable isotope labeling. Finally, the key enzymes of the TCA cycle in tissues and cells were verified by RT-PCR.

**Results:**

Administration of PTX to model mice resulted in neurological damage, shown by both water-maze tests and hippocampal tissue sections. Twenty-four metabolites and five associated metabolic pathways were found to differ significantly between the hippocampal tissues of the model and control groups. These included metabolites and pathways related to the TCA cycle and pyruvate metabolism. Metabolomics analysis using stable isotope labeling showed significant changes in metabolites associated with the TCA cycle compared with the control group (*P* < 0.05). Finally, RT-PCR verified that the expression of key enzymes in the TCA cycle was changed to different degrees in both hippocampal tissues and cells.

**Conclusion:**

Our results showed that PTX neurotoxicity in hippocampal tissue and neuron cells was associated with inhibition of the TCA cycle. This inhibition leads to brain insufficiency and impaired metabolism, resulting in various neurotoxic symptoms.

## 1. Introduction

Chemotherapy-induced neurotoxicity is a serious and persistent side effect that can be caused by many anticancer drugs, such as platinum compounds, taxanes, and vinca alkaloids [1]. Of these, PTX-induced neurotoxicity has been shown to be a dose-limiting toxic side effect. Evidence has shown that 62.5% of patients still had neurotoxic symptoms three months after the end of chemotherapy with PTX and that 47% of women still had neurotoxic symptoms six years after treatment [2]. This seriously affects the quality of life of patients. Therefore, it is of great significance to clarify the mechanism of PTX neurotoxicity and seek new therapeutic targets and directions for clinical practice.

In recent years, there have been numerous studies on the mechanism of PTX-induced neurotoxicity. These have indicated that mitochondrial disorders [3], degeneration of nerve fibers in the epidermis [4], axonal membrane ion channel disorders [5], and neuroinflammation [6] are related to PTX-induced neuropathy. However, the mechanism of action remains unclear. A clear understanding of the mechanism of action can help discover therapeutic agents and thus prevent the condition in advance. Therefore, it is urgent to clarify the mechanism of action. At the same time, after decades of efforts, the treatment of chemotherapy-induced neurotoxicity seems far from satisfactory. Moreover, genome-based targeted studies have not been able to identify suitable drugs for its prevention and treatment [7]. Researchers recognize the need for systematic analysis of the body's response to drug therapy, rather than targeting a single protein or pathway. Therefore, it is important to elucidate metabolic abnormalities from a systematic perspective for understanding disease mechanisms and developing appropriate drugs.

Metabolomics is currently the preferred method for the discovery of biomarkers, and it has a wide range of applications in the study of pathological mechanisms, disease diagnosis, prevention, and treatment [8, 9]. Conventional metabolomics only aims at identifying different metabolites and determination of related pathways to study the mechanism of action, which have some drawbacks, because the analysis may not be able to detect changes in specific metabolic pathways, and the analysis of experimental data often relies on conventional metabolic pathways and easily ignores the possibility of novel pathways. In recent years, stable isotope labeled metabolomics has attracted extensive attention due to its significant advantages in the analysis of metabolic flux and thus the discovery of new metabolic pathways. There has been particular development in the metabolomics of isotope-labeled cells [10, 11], allowing the accurate study of a specific metabolic pathway of a specific metabolite in the metabolic network based on its labeling position and number of isotope atoms in the metabolites.

The tricarboxylic acid cycle (TCA cycle), which takes place in the mitochondria, is the main source of cellular energy and is involved in many metabolic pathways in the cell. Recent studies have shown that dysfunction of enzymes involved in the TCA cycle leading to the inhibition of the pathway may be involved in the development and progression of neurological diseases [12]. The TCA cycle is a ubiquitous metabolic pathway in aerobic organisms and forms a pivotal link between the metabolism of three enzymes and energy metabolism in the body. It has been reported that the *α*-ketoglutarate dehydrogenase complex (KGDHC) is reduced in many neurodegenerative diseases, including Parkinson's disease and Alzheimer's disease [12–14]. Malate dehydrogenase deficiency is associated with early-onset severe encephalopathy [15]. Citrate synthase (CS), as the first key enzyme of the TCA cycle, is essential for maintaining cellular energy production. Although no disease related to its dysfunction has been reported, studies have shown that decreased CS expression can lead to reduced ATP production and increased oxidative damage, which leads to cell death *in vitro* [16].

The present study used a novel combination of *in vivo* and *in vitro* experiments, based on untargeted metabolomics and stable isotope tracer targeted metabolomics, together with multivariate statistical analysis, to explore the relationships between the TCA cycle and its associated enzymes and the development and progression of PTX-induced neurotoxicity. The aim was a comprehensive investigation of the mechanism of PTX-induced neurotoxicity, thus providing new targets and directions for the prevention and treatment of PTX-induced neurotoxicity in clinical applications.

## 2. Materials and Methods

### 2.1. Animal Model

Six to seven-week-old Chinese Kun Ming (KM) mice (male, 35–40 g) were obtained from Jinan Pengyue Experimental Animal Breeding Co., Ltd, China. The animals were housed in automatically controlled conditions, with free access to food and water and a strict 12-hour day and night cycle (lights on from 8 : 00 to 20 : 00). This study not only strictly followed the guidelines of the National Institutes of Health on the care and use of experimental animals but also was approved by the ethics committee of the hospital (No. T20220323006).

After a week of acclimatization, the mice were randomly allocated to the control and PTX treatment groups (*n* = 12 per group). Rats in the PTX treatment group were given 6 mg/kg PTX every three days via intraperitoneal injection for two weeks with a total of five injections. Animals in the untreated control group were given the same volume of normal saline mixed with DMSO in a ratio of 9 : 1. PTX was purchased from Macklin Biochemical (Shanghai, China). The dose of PTX was selected based on the usual clinical treatment dose and earlier reports [17, 18].

### 2.2. Morris Water Maze

The circular pool (diameter 120 cm, height 60 cm) was filled with water at 25 ± 2°C. The maze was divided into four quadrants. The first quadrant contains a transparent escape platform (10 cm in diameter); the platform was located below the surface of the water (no more than 2 cm) and remained unchanged during the experiment. Skim milk powder was added to the water to obscure the platform. In the training experiment, each mouse was placed in the water of a different quadrant and allowed to explore freely for 60 s. If the mouse found the platform within 60 s, the time taken (in seconds) to find the platform was recorded. If the mouse did not find the platform within 60 s, the time was recorded as 60 s and manual guidance while remaining on the platform for 20 s. The mice were trained for five consecutive days before conducting the exploration experiment. During the exploration experiment, the escape platform was removed and each mouse entered the water in the quadrant opposite the platform (i.e., the third quadrant). The number of times the mouse crossed the place where the platform had been and the time it stayed in the target quadrant within 120 s were recorded. The mice were recorded using a CCD camera connected to a computer system. Data were imported into GraphPad Prism 8.0.2 for Student's test and one-way ANOVA.

### 2.3. Hippocampal Tissue Collection

At the end of the drug administration, nine mice in each group were randomly selected for sacrifice by cervical dislocation and placed on ice for dissection for harvesting the hippocampal tissue samples. Part of the tissue samples was fixed with paraformaldehyde, embedded in paraffin, and stained with hematoxylin and eosin (HE) for histopathological evaluation. The remaining tissue was rinsed with phosphate-buffered saline (PBS, pH = 7.2) two to three times. After washing, the samples were rapidly frozen in liquid nitrogen and stored at −80°C for GC-MS metabolite detection and PCR analysis.

### 2.4. Hematoxylin-Eosin (HE) Staining

The hippocampal tissues were fixed, paraffin-embedded, and sectioned. The samples from each group were numbered and placed in the dewatering tank. The sections were dewaxed with xylene and dehydrated in an ethanol gradient. The sections were then stained with HE, dried, sealed with neutral gum, and evaluated.

### 2.5. Untargeted Metabolomics Experiments

Fifty milligrams of tissue were weighed and homogenized, after which methanol was added to extract metabolites. After extraction, the metabolites were derivatized, and the treated samples were analyzed by GC-MS (the QC samples were a mixture of all samples from the experimental and control groups). Firstly, QC samples were collected for analysis, and endogenous compounds were screened for database construction. Samples from both the experimental and control groups were analyzed using the database constructed from the QC samples. The resultant data were normalized and imported into SIMCA-P 14.1 and SPSS 19.0 for statistical analysis, and differential metabolites were screened. The differential metabolites were then introduced into MetaboAnalyst 5.0 for metabolic pathway analysis.

### 2.6. HT-22 Cell Culture

HT-22 cells, derived from mouse hippocampal neuronal cultures, were provided by Shanghai Sig Biotechnology Co., Ltd. The cells were cultured in Dulbecco's Modified Eagle Medium (DMEM, Shanghai Darthill Biotechnology Co., Ltd, Shanghai, China) supplemented with 10% fetal bovine serum (FBS, Biological Industries Israel Beit Haemek Ltd., Shanghai, China) and antibiotics (penicillin-streptomycin solution (100X), Beijing Labgic Technology Co., Ltd., Beijing, China) in a humidified cell incubator with 5% CO_2_ and 95% fresh air at 37°C.

### 2.7. Cell Viability Assay

An MTT assay was used to assess the viability of the neuron cells. After digestion with trypsin-EDTA, cells in the logarithmic growth phase were collected, resuspended, and seeded into 96-well plates. The plates were placed in the incubator for 24 h, and a culture medium containing different concentrations of PTX was added into five parallel wells. Control wells contained culture medium with the same concentration of DMSO drug solvent as the experimental group, while the blank zero-adjustment wells contained only the same volume of medium. The plates were incubated for 48 h. The culture supernatants were then rapidly discarded by inverting the plates, and freshly prepared MTT solution was added. The plates were incubated for further 4 h, after which the medium was carefully removed and DMSO was added before mixing well on a plate shaker. Absorbances were then read at 490 nm on a Multiskan FC microplate reader (Thermo Scientific, USA).

### 2.8. ^13^C-Labeling Experiments and Targeted Metabolomics Analysis

HT-22 cells were grown to approximately 85% confluence before subculturing and dividing into two groups. The cells were allowed to adhere before replacing the medium with a glucose-free medium. After six hours of starvation treatment, the culture medium was replaced with medium containing U-^13^C-glucose. The experimental group was treated with 10 nM PTX, while control cells received the same volume of DMSO as the experimental group. Three replicates of each sample were used. After incubation for 48 h, the cells were collected and metabolites were extracted with 1 ml of 80% methanol. After derivatizing treatment, GC-MS was used to determine the metabolite contents using the selective ion monitoring mode (SIM) quantitative method. The results were expressed as the content of ^13^C labeled with different atomic numbers. “*M*” represented the mass of the unlabeled metabolite, and “*X*” represented the number of labeled carbon atoms in the molecule. The targeted quantitative analysis method followed a previously described protocol [19], and the settings of the specific system parameters are shown in Table S1. Data were collected to analyze the trend of change in the concentration of each metabolite.

### 2.9. Total RNA Extraction and qRT-PCR Analysis

Cells or mouse hippocampal tissue (about 50–100 mg) were collected, and total RNA was first extracted with TRIpure/chloroform, and then quantitative PCR was performed on Bio-rad Cx96 Detection System (Bio-rad, USA) using the SYBR green PCR kit (Applied Biosystems, USA) and gene-specific primers. *β*-Actin was used as the internal control, and the 2^−ΔΔCt^ method was used to normalize expression to those of *β*-actin [20]. The sequences of the primers used for qRT-PCR are provided in Table 1.

## 3. Results

### 3.1. Morris Water Maze

**Figure 1:**
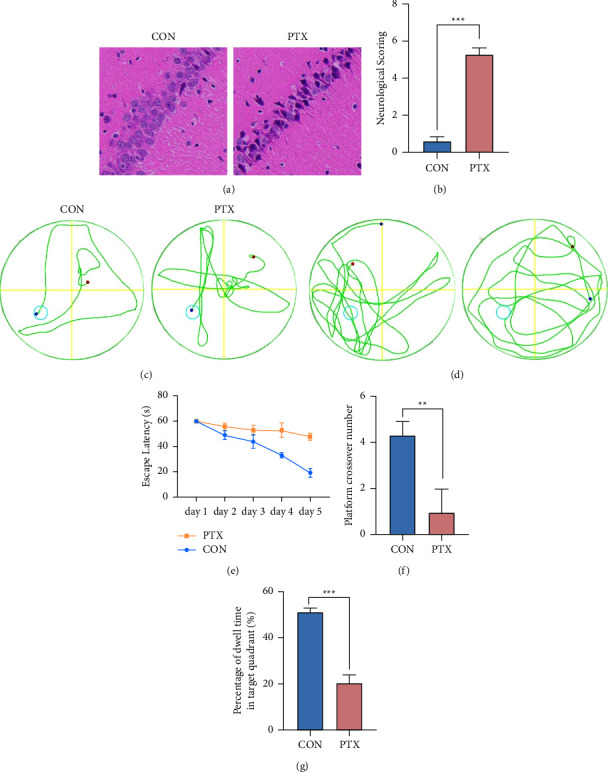
Assessment of the disease activity index and spatial memory and cognition in mice. (a) Histopathological analysis of hippocampal samples from the control and PTX groups. (b) Hippocampal tissues were stained with HE (100x magnification). (c) Representative trajectories of mice in the two groups during the Morris water maze navigation experiment. (d) Exploration of the representative movements of mice in the two groups during the experiment. (e) Average incubation period of escape in mice during navigation experiment. (f) The average number of times the mice passed the platform during the exploration experiment. (g) Exploration of the percentage of time the mice stayed in the target quadrant during the experiment. The blue circle represents the position of the platform, and the green represents the trajectory of the mouse. The data are expressed as the mean ± SD, ^*∗∗*^*p* < 0.01, ^*∗∗∗*^*p* < 0.001 vs. control group.

The Morris water maze was used to evaluate the effects of PTX on the spatial cognition of mice. In the training phase, compared with the control group, the PTX group showed significantly longer escape incubation periods starting from day 3 (*P* < 0.01, Figure 1(e)). In the exploration experiment stage, compared with the control group, the PTX experimental group showed disorganized and irregular trajectories of motion (Figures 1(c) and 1(d)), and the duration of stay in the target quadrant and the number of times of crossing the platform were significantly smaller than those of the control group (*P* < 0.01, Figures 1(f) and 1(g)). These results suggested that PTX can cause spatial cognitive decline in mice.

### 3.2. Histomorphological Observations of Hippocampal Tissue

The neurological severity score (NSS) was calculated by combining the body response and motor ability of the mice. As shown in Figure 1(b), the NSSs in the PTX-treatment group were significantly higher than those in the control group.

HE staining was used to examine the morphological changes in hippocampal tissues under light microscopy, as shown in Figure 1(a). The neuronal morphology in the control group was normal with no evidence of degeneration. In contrast, the PTX group showed nuclear pyknosis, cell differentiation, and decreased cell numbers.

### 3.3. Untargeted Metabolomics Analysis

#### 3.3.1. Statistical Analysis

**Figure 2:**
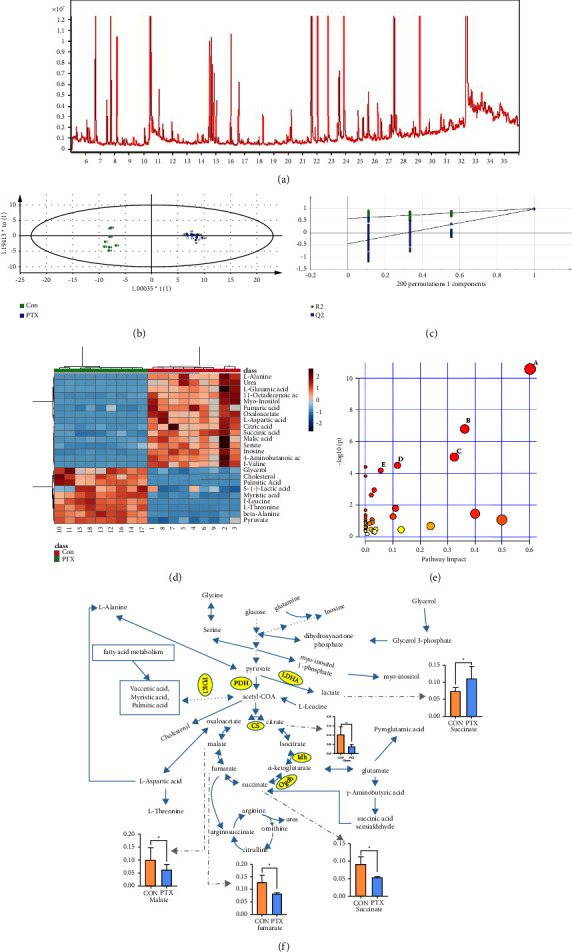
(a) Representative GC-MS total ion current (TIC) chromatograms of hippocampal samples from the mixed PTX and control groups. (b) OPLS-DA score chart in hippocampal tissue. (c) 200 permutation test chart in hippocampal tissue. (d) Heatmap of differentially expressed metabolites in the hippocampal samples from the PTX-treated and control groups. The color of each section is proportional to the significance of the change in metabolites (red, up-regulated; blue, down-regulated). Rows correspond to the samples, and columns correspond to the metabolites. (e) Summary of pathway analysis using MetaboAnalyst 5.0. (A) Alanine, aspartate, and glutamate metabolism, (B) citrate cycle (TCA cycle), (C) pyruvate metabolism, (D) arginine biosynthesis, and (E) glyoxylate and dicarboxylate metabolism. (f) Metabolic pathways of differential metabolites in the hippocampal tissue of PTX-treated mice. The bar chart shows the relative content of metabolites related to glucose as substrate in energy metabolism.

GC-MS was used to analyze the hippocampal tissue samples from different groups. The chromatograms of the hippocampus quality control (QC) samples showed strong signals. Representative total ion chromatograms (TICs) are shown in Figure 2(a).

Multivariate statistical analysis was performed on the original data, and the OPLS-DA method was used to identify the differential metabolites. As can be seen from the OPLS-DA score chart (Figure 2(b)), the experimental group was significantly separated from the control group, indicating significant changes in the levels of endogenous metabolites in the experimental group. In general, in the OPLS-DA model, the closer *R*^2^ and *Q*^2^ are to 1, the more stable the model is. For the OPLS-DA model established in this study, *R*^2^ = 0.997 and *Q*^2^ = 0.976, thus meeting the requirements and indicating that the established model was stable and reliable and could be used for further data analysis. In addition, the validity of the model was confirmed by the substitution test, which showed that the intersection of the blue regression line (*Q*^2^) and the vertical axis (left) was below zero (Figure 2(c)).

We further used MetaboAnalyst 5.0 to investigate metabolic differences between the two groups. Cluster analysis of the differential metabolites in the hippocampus showed the presence of two distinct clusters in most samples, with only a small number of samples overlapping (Figure 2(d)). These results are consistent with those of the OPLS-DA analysis.

#### 3.3.2. Identification of Potential Metabolic Markers

The VIP (VIP>1) and *p* values (*p* < 0.05) used for screening showed significant differences in 24 metabolites between the experimental and control groups. These included mostly amino acid, fatty acid, energy, and lipid metabolites. The specific changes in these metabolites are shown in Table 2, while the relative distribution of each metabolite in the hippocampus is presented in Figure 2(d).

#### 3.3.3. Metabolic Pathway Analysis

MetaboAnalyst 5.0 was used to analyze the pathways associated with the differential metabolites between the control and PTX groups. We identified five significant metabolic pathways (raw *p* < 0.05, impact >0). These pathways included “alanine, aspartate, and glutamate metabolism,” “citrate cycle (TCA cycle),” “pyruvate metabolism,” “arginine biosynthesis,” and “glyoxylate and dicarboxylate metabolism.” These pathways can be found in the Kyoto Encyclopedia of Genes and Genomes (KEGG; https://www.kegg.jp). The details of the pathway analysis are shown in Table 3 and Figure 2(e), and a summary is shown in Figure 2(f).

#### 3.3.4. MTT Assays

**Figure 3:**
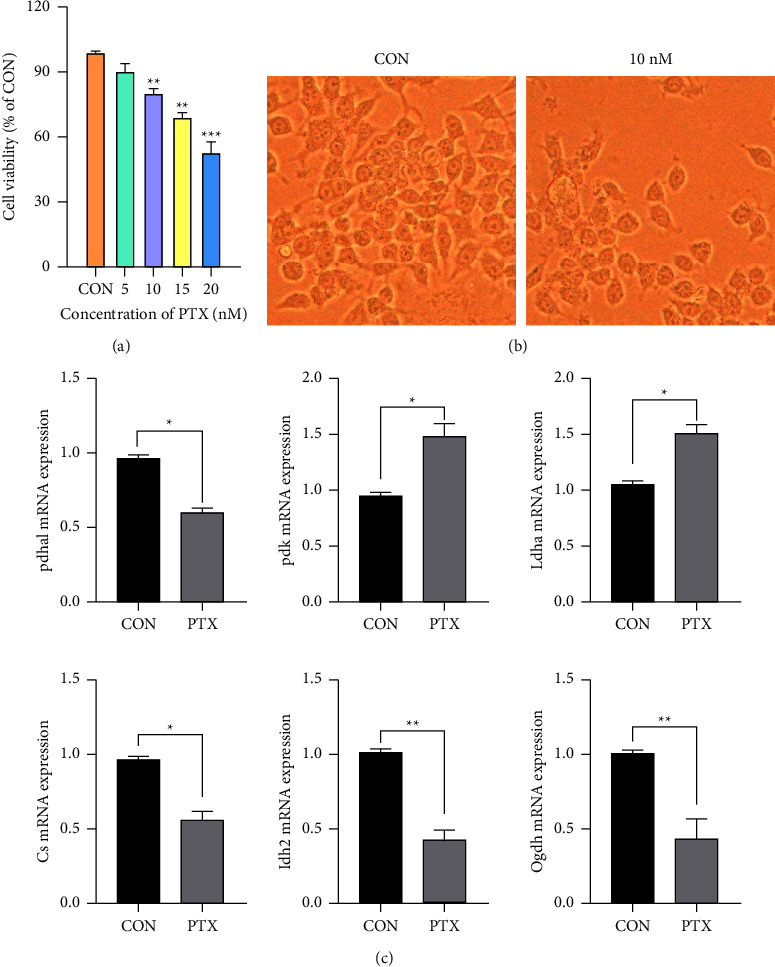
Neurotoxicity in HT-22 cells after PTX treatment. (a) Cell viability after treatment with varying concentrations of PTX. (b) Representative light micrographs showing cell morphology in the different experimental groups (100x magnification). (c) Relative mRNA expression of key enzymes associated with glucose metabolism in HT-22 cells, measured by qRT-PCR. The data were compared using Student's *t*-tests, ^*∗*^*p* < 0.05, ^*∗∗*^*p* < 0.01.

We used MTT assays, together with the findings of previous reports, to select the appropriate drug concentrations, investigating the effects of different concentrations of PTX on the viability of HT-22 cells, as shown in Figure 3(a). The viability of cells decreased significantly at a PTX concentration of 20 nM, while at 10 nM, the cell viability was still over 85%, although less than that seen at 5 nM. Thus, 10 nM was chosen as the final PTX concentration.  Figure 3(b) shows the histology of the cells after 48-h of culture. Compared with the control group, the numbers of PTX-treated cells were not only reduced, but the neurons appeared differentiated with an absence of connecting synapses.

#### 3.3.5. Stable Isotope-Assisted Targeted Metabolomics Analysis

Based on the results of the *in vivo* studies, we then established a quantitative analysis method for the selective ion monitoring model of important glucose-related metabolites. Using stable isotope-assisted targeted metabolomics *in vitro*, U-^13^C-glucose was used to label intracellular metabolites and trace the specific changes in glucose metabolism in neurons. This showed that, compared with the control group, the *M* + 3 lactate was significantly reduced in the PTX treatment group, while no differences were seen in the *M* + 0 lactate. The citrate contents citrate in *M* + 0, *M* + 2, and *M* + 4 were also significantly reduced, as were the contents of succinate and malate in *M* + 2 and *M* + 4. These results suggest inhibition of the intracellular TCA cycle and activation of glycolysis.

#### 3.3.6. RT-PCR in Tissue and Cells

**Figure 4:**
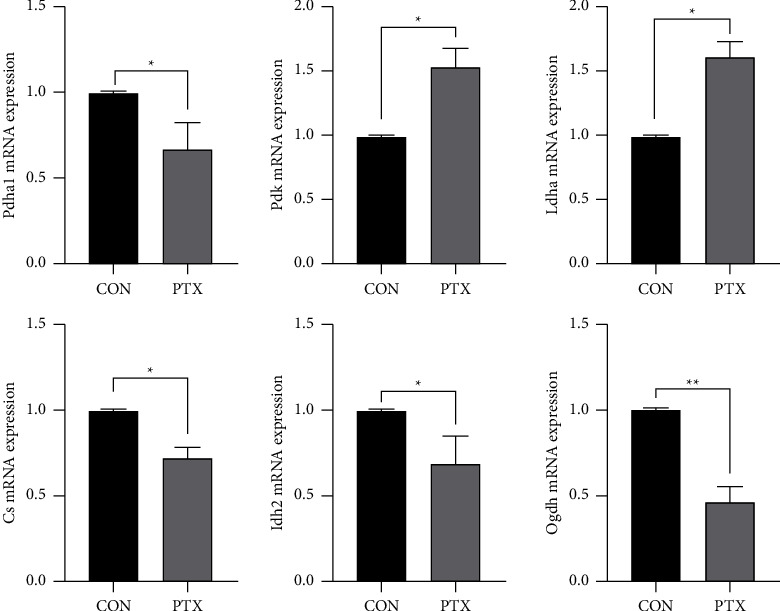
The relative mRNA levels of key enzymes associated with glucose metabolism in the mouse hippocampus as measured by qRT-PCR. The data were compared using Student's *t*-tests, ^*∗*^*p* < 0.05, ^*∗∗*^*p* < 0.01.

We detected and analyzed the expression of Pdha1, Pdk1, Ldha, Cs, Idh2, and Ogdh in mouse hippocampal tissue and neuron cells using RT-PCR (Figure 4 and 3(c)). Compared with the control group, the expression of Pdha1, Cs, Idh2, and Ogdh was reduced to varying degrees in the PTX group, while the expression of Pdk and Ldha was increased (*P* < 0.05).

## 4. Discussion

As is well known, the mitochondria are the primary source of energy in the cell. After the hypothesis linking mitochondrial dysfunction and neurological diseases was proposed, researchers focused on the relationship between mitochondrial energy supply and Alzheimer's disease [21], Parkinson's disease [22], depression [23], and epilepsy [24] to understand the pathogenesis of various diseases and have made many important discoveries. In addition, drugs that treat mitochondrial disorders have been found to relieve the symptoms of many of these diseases [25]. However, the specific mechanism still requires clarification. Therefore, it is suggested that investigation of the substrate supply for energy metabolism may provide further evidence for the hypothesis of mitochondrial dysfunction.

Disordered energy metabolism is associated with diseases of the nervous system [26, 27]. A lack of energy substrates will lead to a series of biochemical changes. Glucose metabolism is one of the most important processes involved in the maintenance of the body's energy balance. Glucose metabolism provides 95% or more of the brain's energy needs [28]. In addition, a lack of energy substrates forces the brain to make adaptive changes in its use of glucose. It is estimated that about 70–80% of this brain energy is consumed by neurons and the rest by glial cells [29]. Meanwhile, ATP required by neurons is mainly produced in the mitochondria through oxidative phosphorylation by the TCA cycle. Additional ATP is produced by aerobic glycolysis in the cytoplasm and is necessary to support the high energy demands of synaptic transmission [30]. Therefore, the supply of energy substrates represents an important indicator of disturbances in brain energy metabolism in patients with neurological diseases. This indicates that our prior hypothesis, as well as the conducted research, is meaningful.

The concept of the presence of reduced brain energy metabolism before the diagnosis of neurodegenerative disease was first proposed many years ago [31, 32]. Since then, increasing evidence has shown that impaired brain energy is involved in the development and progression of neurodegenerative diseases [31, 32]. These diseases primarily include Alzheimer's disease [33], Parkinson's disease [34], Huntington's disease [35], frontotemporal dementia (FTD) [36], and amyotrophic lateral sclerosis (ALS) [37]. These diseases are characterized by decreased glucose uptake by neurons, decreased TCA activity, decreased mitochondrial function, and decreased energy support from astrocytes and oligodendrocytes [38, 39]. In addition, neuroinflammation leads to increased glucose consumption by microglia, which sucks energy away from neurons [40]. However, many positron emission tomography (PET) studies have found that these metabolic disorders may occur in different brain regions in different diseases [41]. For example, FTD is mainly associated with the frontal lobe, striatum, and hypothalamus, while ALS mainly occurs in the cortex and spinal cord [11]. Therefore, further identification of areas of impaired glucose metabolism in the brain could be used to better distinguish between various neurodegenerative diseases. In addition, studies have also reported that impaired brain energy is associated with the development of other neurological diseases, including epilepsy [42], depression [27], and schizophrenia [43]. Consistent with previous reports, our results showed that TCA metabolism was significantly impaired in a paclitaxel-induced neurotoxicity mouse model.

**Figure 5:**
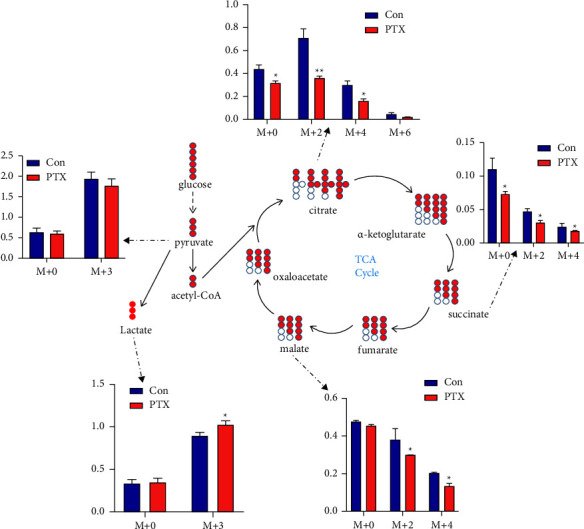
Effects of paclitaxel administration on tricarboxylic acid cycle metabolism in HT-22 cells, shown by changes in metabolite levels. The data were compared using Student's *t*-tests, ^*∗*^*p* < 0.05, ^*∗∗*^*p* < 0.01.

In our study, we combined *in vitro* and *in vivo* studies. *In vivo*, we used an untargeted metabolomics approach to analyze the changes in metabolite levels and metabolic pathways in the hippocampus of mice with PTX-induced neurotoxicity. Combined with multivariate statistical methods, 24 metabolites with statistical differences and 9 related metabolic pathways were identified (Tables 2 and 3). The metabolites associated with energy metabolism included lactic acid, citric acid, fumarate, and succinate, and the metabolic pathways involved included the TCA cycle and pyruvate metabolism. *In vitro*, stable isotope-assisted targeted metabolomics was used to analyze changes in the major metabolites associated with glucose metabolism in the hippocampal neuron cells of PTX-treated mice. The energy metabolism substrate was replaced with U-^13^C glucose to accurately elucidate specific metabolic pathways associated with glucose catabolism disorders based on the isotopic distributions of key metabolites. It was found that the *M*+3 isotopes of lactate were increased, while the *M* + 2 and *M* + 4 isotopes of metabolites in the TCA cycle were decreased after the uptake of fully labeled ^13^C glucose in mouse hippocampal neuron cells after PTX exposure (Figure 5). In addition, the mRNA levels of key enzymes related to TCA metabolism were analyzed in both hippocampal tissue and neuron cells using RT-PCR. The results showed that the expression of pyruvate dehydrogenase kinase (Pdk) was increased (Figures 4 and 3(c)). It has been reported that activation of Pdk can reduce the phosphorylation of pyruvate dehydrogenase (Pdha), followed by inhibition of the conversion of pyruvate to acetyl-CoA in the TCA cycle, leading to the activation of the glycolytic pathway of pyruvate [44]. This explains the observed increased expression of lactate dehydrogenase (Ldha). At the same time, the expression of citrate synthase (Cs), isocitrate dehydrogenase (Idh2), and *α*-ketoglutarate dehydrogenase (Ogdh), the key enzymes in the TCA cycle, were reduced to varying degrees (Figures 4 and 3(c)). Cs is the first rate-limiting enzyme in the TCA cycle. Although the decreased expression of Cs has not been reported to be associated with any specific diseases, several studies have shown that reduced Cs expression can lead to decreased ATP production and increased oxidative damage, leading to cell apoptosis [16, 45, 46]. In comparison, there have been many studies on the associations between Idh and Ogdh with diseases, including neuroglioma, leukemia, nervous system metabolic disorders, and degenerative diseases of the nervous system [13, 47, 48]. Meanwhile, our results showed that the expression of both Idh and Ogdh was decreased to varying degrees, which was consistent with the results of studies on these two enzymes in different neurodegenerative diseases. Taken together, this study may provide direct metabolic evidence for the inhibition of TCA cycle metabolism in the mouse hippocampus after PTX treatment.

In conclusion, we suggest that mitochondrial pyruvate carriers and acetyl-CoA could be potential targets for the treatment of PTX-induced neurotoxicity. At the same time, the results of the study showed that it was possible to accurately elucidate the mechanisms underlying PTX neurotoxicity by combining stable isotope catabolic analysis with an analysis of mitochondrial structure and function. This study provides unique insights into the mechanism responsible for PTX-mediated neurotoxicity, the discovery of new targets, and the development of new therapeutic agents.

Nevertheless, the study has some limitations. Firstly, the metabolomics approaches using stable isotope tracers have only been studied *in vitro*, and the results may ignore potential changes and perturbations present in metabolism *in vivo*. We can further apply this stable isotope approach to *in vivo* studies. At the same time, the quantitative detection of other metabolites associated with glucose metabolism should be performed to further explain the fluxes in glucose metabolism. Secondly, the effects of PTX on other parts of the brain, such as the cerebral cortex, striatum, and hypothalamus, should be elucidated separately to better understand the mechanism of PTX-induced neurotoxicity. In addition, it would be necessary to further clarify the expression of proteins and genes in the relevant pathways in the different parts of the brain affected by PTX.

## Figures and Tables

**Table 1:** Sequences of primers used for *r* qRT-PCR.

Gene name	Forward primer (5′-3′)	Reverse primer (5′-3′)
Pdha1	CATAGGAATGCTGGTGGCTCTGTG	TCTCAGGTTCAGGGTGGCAAGG
Pdk1	TTAGAGGGCTACGGGACAGATGC	GTAATGCTTCCAGGCGGCTTTATTG
Ldha	CGCAGACAAGGAGCAGTGGAAG	ACCCGCCTAAGGTTCTTCATTATGC
Cs	CCTGTGGTCTTCCTGGTCGTTTG	CTGGTCATTCCTGAGCCCTTGTTC
Idh2	ATCTGGCAGTTCATCAAGGAGAAGC	ATTGGTCTGGTCACGGTTTGGAAG
Ogdh	CCAGGAAGAGCACAAGAACCAAGG	GACAGGCTTAGCACGGTCAATGG
*β*-Actin	AGAGGGAAATCGTGCGTGAC	CAATAGTGATGACCTGGDDGT

**Table 2:** List of metabolites in hippocampal tissues that differed significantly between the PTX and the control groups.

Metabolites	HMDB	VIP	*P* value	Fold change	Trend
Vaccenic acid	HMDB0003231	1.182	3.916*E*–5	0.065	↓
Gamma-aminobutyric acid	HMDB0000112	1.207	4.128*E*–5	0.083	↓
Beta-alanine	HMDB0000056	1.240	9.222*E*–8	1.118	↑
Cholesterol	HMDB0000067	1.147	1.416*E*–5	3.649	↑
D-lactic acid	HMDB0001311	1.217	1.316*E*–5	1.431	↑
Citric acid	HMDB0000094	1.113	1.285*E*–3	0.105	↓
Fumaric acid	HMDB0000134	1.189	1.980*E*–4	0.094	↓
Glycerol	HMDB0000131	1.088	5.146*E*–5	5.160	↑
Oxaloacetate	HMDB0000223	1.235	4.864*E*–6	0.084	↓
L-glutamic acid	HMDB0000148	1.167	2.174*E*–3	0.067	↓
Inosine	HMDB0000195	1.238	1.580*E*–6	0.094	↓
Pyruvate	HMDB0000243	1.152	6.934*E*–5	1.024	↑
L-Alanine	HMDB0000161	1.180	6.416*E*–5	0.018	↓
L-aspartic acid	HMDB0000191	1.195	1.821*E*–5	0.006	↓
L-Leucine	HMDB0000687	1.259	3.373*E*–7	1.057	↑
L-Threonine	HMDB0000167	1.261	3.016*E*–8	1.071	↑
L-valine	HMDB0000883	1.185	7.210*E*–5	0.026	↓
Malic acid	HMDB0000744	1.122	4.191*E*–4	0.029	↓
Myo-inositol	HMDB0000211	1.149	1.830*E*–4	0.034	↓
Myristic acid	HMDB0000806	1.260	3.091*E*–8	5.978	↑
Palmitic acid	HMDB0000220	1.166	7.201*E*–6	5.187	↑
Serine	HMDB0062263	1.153	6.415*E*–6	0.105	↓
Succinic acid	HMDB0000254	1.185	4.194*E*–5	0.408	↓
Urea	HMDB0000294	1.122	1.729*E*–4	0.025	↓

HMDB: The human metabolome database, fold change: PTX/control, and VIP: variable influence on projection.

**Table 3:** Metabolic pathways significantly associated with PTX-mediated neurotoxicity, from MetaboAnalyst 5.0 analysis.

Pathway name	Match status	Raw *p*	Impact
Alanine, aspartate and glutamate metabolism	9/28	2.759*E*–11	0.601
Citrate cycle (TCA cycle)	6/20	1.585*E*–7	0.361
Pyruvate metabolism	5/22	9.200*E*–6	0.323
Arginine biosynthesis	4/14	3.115*E*–5	0.117
Glyoxylate and dicarboxylate metabolism	5/32	6.395*E*–5	0.056

Raw *P* value < 0.05 and Impact >0 were considered to have significant differences in metabolic pathways.

## Data Availability

The datasets used and/or analyzed during the current study are available from the corresponding author on reasonable request.

## Supplementary Materials

Supplementary Materials Supplementary data include the instruments used in the GC-MS analysis for the determination of the five compounds in the TCA cycle as well as the corresponding parameters.

## References

[1] Was H., Borkowska A., Bagues A. (2022). Mechanisms of chemotherapy-induced neurotoxicity. *Frontiers in Pharmacology*.

[2] Winters-Stone K. M., Horak F., Jacobs P. G. (2017). Falls, functioning, and disability among women with persistent symptoms of chemotherapy-induced peripheral neuropathy. *Journal of Clinical Oncology*.

[3] Xiao W. H., Zheng H., Bennett G. J. (2012). Characterization of oxaliplatin-induced chronic painful peripheral neuropathy in the rat and comparison with the neuropathy induced by paclitaxel. *Neuroscience*.

[4] Wozniak K. M., Vornov J. J., Wu Y. (2018). Peripheral neuropathy induced by microtubule-targeted chemotherapies: insights into acute injury and long-term recovery. *Cancer Research*.

[5] Xiao W., Boroujerdi A., Bennett G. J., Luo Z. D. (2007). Chemotherapy-evoked painful peripheral neuropathy: analgesic effects of gabapentin and effects on expression of the alpha-2-delta type-1 calcium channel subunit. *Neuroscience*.

[6] Brandolini L., d’Angelo M., Antonosante A., Cimini A., Allegretti M. (2019). Chemokine signaling in chemotherapy-induced neuropathic pain. *International Journal of Molecular Sciences*.

[7] Al-Mahayri Z. N., AlAhmad M. M., Ali B. R. (2021). Current opinion on the pharmacogenomics of paclitaxel-induced toxicity. *Expert Opinion on Drug Metabolism and Toxicology*.

[8] Dang R., Yang M., Cui C. (2021). Activation of angiotensin-converting enzyme 2/angiotensin (1-7)/mas receptor axis triggers autophagy and suppresses microglia proinflammatory polarization via forkhead box class O1 signaling. *Aging Cell*.

[9] Geng C., Guo Y., Qiao Y. (2019). UPLC-Q-TOF-MS profiling of the hippocampus reveals metabolite biomarkers for the impact of Dl-3-n-butylphthalide on the lipopolysaccharide-induced rat model of depression. *Neuropsychiatric Disease and Treatment*.

[10] Cuperlović-Culf M., Barnett D. A., Culf A. S., Chute I. (2010). Cell culture metabolomics: applications and future directions. *Drug Discovery Today*.

[11] Ahmed R. M., Irish M., Piguet O. (2016). Amyotrophic lateral sclerosis and frontotemporal dementia: distinct and overlapping changes in eating behaviour and metabolism. *The Lancet Neurology*.

[12] Kang W., Suzuki M., Saito T., Miyado K. (2021). Emerging role of TCA cycle-related enzymes in human diseases. *International Journal of Molecular Sciences*.

[13] Gibson G. E., Blass J. P., Beal M. F., Bunik V. (2005). The alpha-ketoglutarate-dehydrogenase complex: A mediator between mitochondria and oxidative stress in neurodegeneration. *Molecular Neurobiology*.

[14] Gibson G. E., Park L. C., Sheu K. F., Blass J. P., Calingasan N. Y. (2000). The alpha-ketoglutarate dehydrogenase complex in neurodegeneration. *Neurochemistry International*.

[15] Broeks M. H., Shamseldin H. E., Alhashem A. (2019). MDH1 deficiency is a metabolic disorder of the malate-aspartate shuttle associated with early onset severe encephalopathy. *Human Genetics*.

[16] Cai Q., Zhao M., Liu X. (2017). Reduced expression of citrate synthase leads to excessive superoxide formation and cell apoptosis. *Biochemical and Biophysical Research Communications*.

[17] Toma W., Kyte S. L., Bagdas D. (2017). Effects of paclitaxel on the development of neuropathy and affective behaviors in the mouse. *Neuropharmacology*.

[18] Toma W., Caillaud M., Patel N. H. (2021). N-acylethanolamine-hydrolysing acid amidase: a new potential target to treat paclitaxel-induced neuropathy. *European Journal of Pain*.

[19] Zhou Y., Song R., Zhang Z. (2016). The development of plasma pseudotargeted GC-MS metabolic profiling and its application in bladder cancer. *Analytical and Bioanalytical Chemistry*.

[20] Livak K. J., Schmittgen T. D. (2001). Analysis of relative gene expression data using real-time quantitative PCR and the 2−ΔΔCT method. *Methods*.

[21] Potenza M. A., Sgarra L., Desantis V., Nacci C., Montagnani M. (2021). Diabetes and Alzheimer’s disease: might mitochondrial dysfunction help deciphering the common path?. *Antioxidants*.

[22] Gao X. Y., Yang T., Gu Y., Sun X. H. (2022). Mitochondrial dysfunction in Parkinson’s disease: from mechanistic insights to therapy. *Frontiers in Aging Neuroscience*.

[23] Bansal Y., Kuhad A. (2016). Mitochondrial dysfunction in depression. *Current Neuropharmacology*.

[24] McDonald T. S., Carrasco-Pozo C., Hodson M. P., Borges K. (2017). Alterations in cytosolic and mitochondrial [U-^13^C]glucose metabolism in a chronic epilepsy mouse model. *eNeuro*.

[25] Hurko O. (2013). Drug development for rare mitochondrial disorders. *Neurotherapeutics*.

[26] Chen Z., Zhong C. (2013). Decoding Alzheimer’s disease from perturbed cerebral glucose metabolism: implications for diagnostic and therapeutic strategies. *Progress in Neurobiology*.

[27] Ling-Hu T., Liu S. B., Gao Y., Han Y. M., Tian J. S., Qin X. M. (2021). Stable isotope-resolved metabolomics reveals the abnormal brain glucose catabolism in depression based on chronic unpredictable mild stress rats. *Journal of Proteome Research*.

[28] Dienel G. A. (2019). Brain glucose metabolism: integration of energetics with function. *Physiological Reviews*.

[29] Hyder F., Rothman D. L., Bennett M. R. (2013). Cortical energy demands of signaling and nonsignaling components in brain are conserved across mammalian species and activity levels. *Proceedings of the National Academy of Sciences of the USA*.

[30] Bordone M. P., Salman M. M., Titus H. E. (2019). The energetic brain-a review from students to students. *Journal of Neurochemistry*.

[31] Zilberter Y., Zilberter M. (2017). The vicious circle of hypometabolism in neurodegenerative diseases: ways and mechanisms of metabolic correction. *Journal of Neuroscience Research*.

[32] Camandola S., Mattson M. P. (2017). Brain metabolism in health, aging, and neurodegeneration. *The EMBO Journal*.

[33] Butterfield D. A., Halliwell B. (2019). Oxidative stress, dysfunctional glucose metabolism and Alzheimer disease. *Nature Reviews Neuroscience*.

[34] Matthews D. C., Lerman H., Lukic A. (2018). FDG PET Parkinson’s disease-related pattern as a biomarker for clinical trials in early stage disease. *NeuroImage: Clinic*.

[35] Liot G., Valette J., Pépin J., Flament J., Brouillet E. (2017). Energy defects in Huntington’s disease: why“in vivo”evidence matters. *Biochemical and Biophysical Research Communications*.

[36] Lau D. H. W., Hartopp N., Welsh N. J. (2018). Disruption of ER-mitochondria signalling in fronto-temporal dementia and related amyotrophic lateral sclerosis. *Cell Death & Disease*.

[37] Vandoorne T., De Bock K., Van Den Bosch L. (2018). Energy metabolism in ALS: an underappreciated opportunity?. *Acta Neuropathologica*.

[38] Briston T., Hicks A. R. (2018). Mitochondrial dysfunction and neurodegenerative proteinopathies: mechanisms and prospects for therapeutic intervention. *Biochemical Society Transactions*.

[39] Murphy M. P., Hartley R. C. (2018). Mitochondria as a therapeutic target for common pathologies. *Nature Reviews Drug Discovery*.

[40] Aldana B. I. (2019). Microglia-specific metabolic changes in neurodegeneration. *Journal of Molecular Biology*.

[41] Wilson H., Pagano G., Politis M. (2019). Dementia spectrum disorders: lessons learnt from decades with PET research. *Journal of Neural Transmission*.

[42] McDonald T., Hodson M. P., Bederman I., Puchowicz M., Borges K. (2020). Triheptanoin alters [U-^13^C_6_]-glucose incorporation into glycolytic intermediates and increases TCA cycling by normalizing the activities of pyruvate dehydrogenase and oxoglutarate dehydrogenase in a chronic epilepsy mouse model. *Journal of Cerebral Blood Flow and Metabolism*.

[43] Bubber P., Hartounian V., Gibson G. E., Blass J. P. (2011). Abnormalities in the tricarboxylic acid (TCA) cycle in the brains of schizophrenia patients. *European Neuropsychopharmacology*.

[44] Sugden M. C., Holness M. J. (2003). Recent advances in mechanisms regulating glucose oxidation at the level of the pyruvate dehydrogenase complex by PDKs. *American Journal of Physiology. Endocrinology and Metabolism*.

[45] Fišar Z., Hansíková H., Křížová J. (2019). Activities of mitochondrial respiratory chain complexes in platelets of patients with Alzheimer’s disease and depressive disorder. *Mitochondrion*.

[46] Fišar Z., Hroudová J., Hansíková H. (2016). Mitochondrial respiration in the platelets of patients with Alzheimer’s disease. *Current Alzheimer Research*.

[47] Martínez-Reyes I., Chandel N. S. (2020). Mitochondrial TCA cycle metabolites control physiology and disease. *Nature Communications*.

[48] Raimundo N., Baysal B. E., Shadel G. S. (2011). Revisiting the TCA cycle: signaling to tumor formation. *Trends in Molecular Medicine*.

