# Supplementary material for: Paclitaxel Induces Neurotoxicity by Disrupting Tricarboxylic Acid Cycle Metabolic Balance in the Mouse Hippocampus
Source: J Toxicol. 2023 Aug 5;2023:5660481. doi: 10.1155/2023/5660481 (PMC10423086; doi:10.1155/2023/5660481)
Supplement: Supplementary Materials — Supplementary data include the instruments used in the GC-MS analysis for the determination of the five compounds in the TCA cycle as well as the corresponding parameters. [file 5660481.f1.docx]

**GC-MS Analysis**

The samples were analyzed by gas chromatography (Agilent 7890B)/mass spectrometry (Agilent 7000C). The injection volume was set to 1 μL and the shunt ratio was 1:5. The column was HP-5MS (30 m×250 µm×0.25 µm, Agilent J&W Scientific, Folsom, CA, USA). Helium was used as the carrier gas and the flow rate was 1.2 mL/min. The inlet temperature, transmission line temperature, and ion source temperature were 300℃, 280℃, and 230℃, respectively. The solvent delay was 5.5 min, and the detection voltage was 1650 V. The GC heating procedure was as follows: the initial temperature was 70℃ which was maintained for 1 min, then increased to 190℃ at 5℃/min, increased to 310℃ at 15℃/min and held 310℃ for 5 min. Using electron impact ionization (70 eV), 20 spectra/s were obtained in the MS setting. The mass/charge (m/z) range was from 50 to 800. The specific parameters are shown in Table 1.

**Table 1.** Methodological parameters

| metabolites | Retention time（min） | Quantitative ion（m/z） | Qualitative ion（m/z） | linearity range（ng/ml） | R^2^ | LOD（ng/ml） |
| --- | --- | --- | --- | --- | --- | --- |
| Pyruvate | 6.69 | 174 | 115 | 8.00-200000 | 0.9977 | 0.17 |
| Lactate | 6.96 | 190 | 219 | 100-1000000 | 0.9903 | 44.55 |
| succinic acid-d_4_ | 13.27 | 176 | 251 | ---- | ---- |  |
| Succinate | 13.33 | 172 | 247 | 8.00-200000 | 0.9995 | 0.18 |
| Malate | 17.87 | 233 | 245 | 8.00-200000 | 0.9992 | 0.54 |
| Citrate | 25.36 | 273 | 363 | 8.00-200000 | 0.9956 | 0.46 |
